# Successful Treatment of Intracranial Hemorrhage with Recombinant Activated Factor VII in a Patient with Newly Diagnosed Acute Myeloid Leukemia: A Case Report and Review of the Literature

**DOI:** 10.3389/fonc.2015.00029

**Published:** 2015-02-11

**Authors:** Naveen Pemmaraju, Koji Sasaki, Daniel Johnson, Naval Daver, Vahid Afshar-Kharghan, Merry Chen, Sairah Ahmed, Rivka R. Colen, Michael Kwon, Yang Huh, Gautam Borthakur

**Affiliations:** ^1^Department of Leukemia, MD Anderson Cancer Center, Houston, TX, USA; ^2^Department of Internal Medicine, Louisiana State University, New Orleans, LA, USA; ^3^Department of Benign Hematology, MD Anderson Cancer Center, Houston, TX, USA; ^4^Department of Neuro-Oncology, MD Anderson Cancer Center, Houston, TX, USA; ^5^Department of Stem Cell Transplantation, MD Anderson Cancer Center, Houston, TX, USA; ^6^Department of Diagnostic Radiology, MD Anderson Cancer Center, Houston, TX, USA; ^7^Department of Hematopathology, MD Anderson Cancer Center, Houston, TX, USA

**Keywords:** recombinant activated factor VII, acute myeloid leukemia, intracranial hemorrhage

## Abstract

Intracranial hemorrhage (ICH) is a common complication in acute myeloid leukemia (AML) patients with an incidence rate of 6.3% (1). Bleeding disorders related to disseminated intravascular coagulation (DIC) are common complications in AML cases (2). Recombinant activated Factor VII [rFVIIa (NovoSeven^®^)] is approved for the treatment of bleeding complications with FVIII or FIX inhibitors in patients with congenital FVII deficiency. Use of rFVIIa for the treatment of acute hemorrhage in patients without hemophilia has been successful (3, 4). Herein, we describe the successful use of rFVIIa in a patient with acute ICH in the setting of newly diagnosed AML.

A 26-year-old man without prior personal or family history of bleeding disorders who had recently returned from serving in the National Guard in Afghanistan presented with a sore throat, ecchymoses on his shin and thigh, and tongue petechiae. Infectious disease workup was negative. However, complete blood count revealed a white blood cell count of 300,000/μL, and the patient treatment was initiated with hydroxyurea. Upon transfer to our institution for further management, his white blood cell count was 169,800/μL, hemoglobin was 6.8 g/dL, and platelet count was 21,000/μl. The peripheral blood smear was consistent with acute myeloid leukemia (AML), and he was initiated on cytarabine and hydroxyurea. The work up for acute promyelocytic leukemia (APL) including *PML-RARA* fusion transcript analysis by qualitative reverse-transcription polymerase chain reaction was negative and cytogenetics revealed 46 XY, *t*(9;11) (p22;q23).

The patient concomitantly experienced intermittent headaches at presentation. Neuroimaging demonstrated acute multi-focal intra-cerebral hemorrhages. The clinical course, the progression of intra-cerebral hemorrhages, and supportive treatments were summarized (Figure 1).

**Figure 1:**
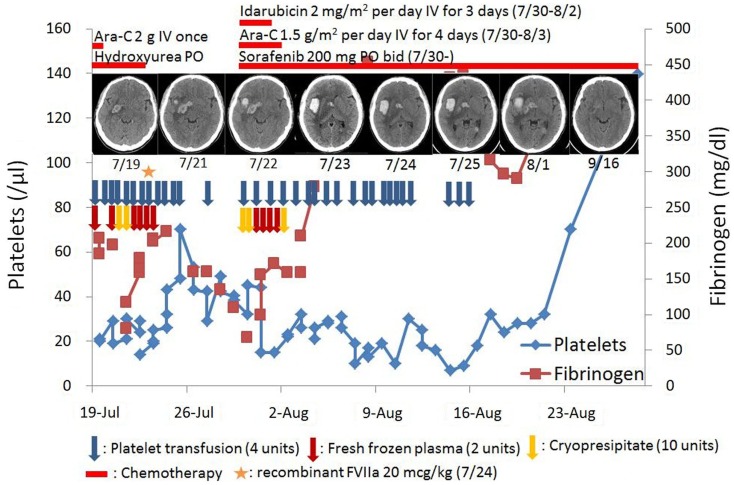
**Clinical course**.

The laboratory evaluation were consistent with DIC: thrombocytopenia (platelet count, 21,000/μL), prolonged prothrombin time (17.7 s), elevated activated partial thromboplastin time (37.4 s), decreased fibrinogen level (184 mg/dL), and an elevated D-dimer (>20 μg/mL). Over the next several days the patient received several units of blood products including 20 units cryoprecipitate, 12 units of fresh frozen plasma, and 48 units of pooled platelet. However, the patient became increasingly somnolent and experienced progressive left-sided weakness. A repeat of neuroimaging revealed increase in the size of his right intra-cerebral hemorrhage (Figure 2). Progression of the patient’s intracranial hemorrhage (ICH) in spite of frequent transfusions with blood products, prompted initiation of treatment with continuous intravenous infusion of aminocaproic acid (1 g/h) and one dose of rFVIIa (20 μg/kg). Within 1 day after infusion of rFVIIa, his ICH stabilized as was evident in his follow-up brain imaging. The patient clinically improved over the subsequent few days with an eventual complete neurologic recovery.

**Figure 2:**
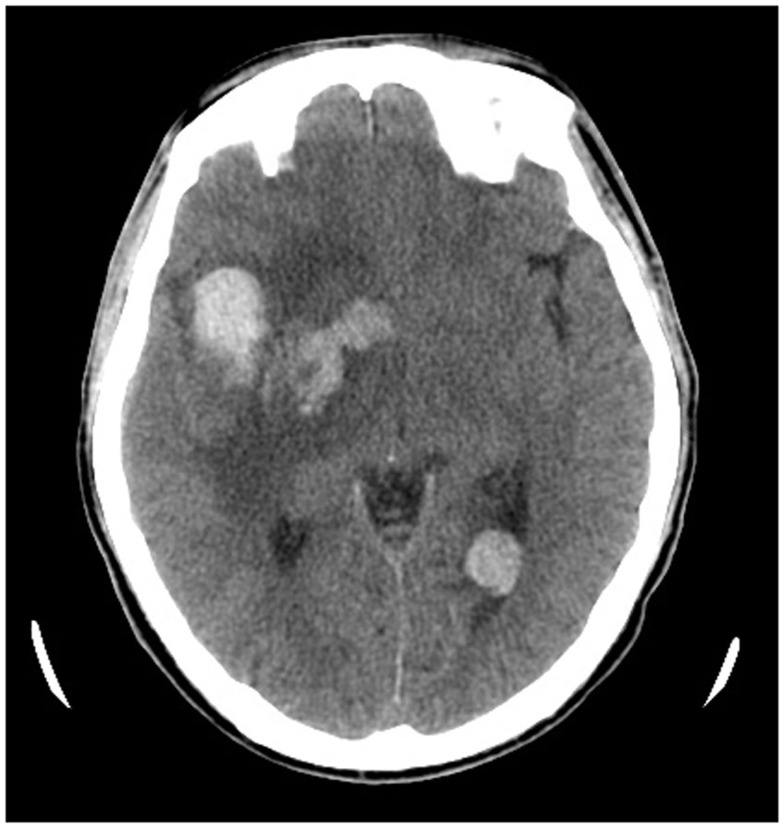
**Computed tomography (CT) scan of head without intravenous (IV) contrast demonstrating scattered multifocal acute intra-cerebral hemorrhage (ICH) and marked perilesional edema after supportive treatment of ICH**.

Finalized bone marrow evaluation of the patient revealed a diagnosis of AML-M5 with *t*(9;11) translocation and a FLT3-ITD mutation. After undergoing induction chemotherapy with idarubicin, cytarabine, and sorafenib and two subsequent cycles of consolidation chemotherapy, he achieved complete morphological remission and had no evidence of molecular minimal residual disease by multiplanar flow-cytometry. Due to the nature of his high risk AML characterized by a FLT3-ITD mutation, he was referred for stem cell transplantation. He has recently undergone an allogeneic matched unrelated donor stem cell transplant using a conditioning regimen of busulfan, fludarabine, clofarabine, and anti-thymoglobulin. The patient has achieved successful engraftment with 100% chimerism, negative minimal residual disease and negative FLT3 mutation, and is participating in a post-stem cell transplant azacitidine maintenance trial. He remains in complete remission 8 months after his initial diagnosis, without further complications or recurrence of ICH.

Although APL has the strongest association with DIC, ICH remains an important cause of early death in all patients with AML. Both monocytic differentiation and hyperleukocytosis are independent risk factors for early death associated with this complication in patients with AML (5).

There is conflicting evidence supporting the early initiation of rFVIIa in patients with ICH, whereas early initiation of rFVIIa was associated with shrinkage of the hematoma, reduction in mortality, and improved functional outcomes at 90 days; it is also associated with a slightly increased risk of thromboembolic complications in patients who received higher dose of rFVIIa (80 μg/kg), and no increased risk of thrombosis in patients who received lower dose of rFVIIa (20 μg/kg) compared to placebo (6). However, subsequent studies have revealed conflicting evidence regarding the effectiveness of early rFVIIa use, confirming reduced hematoma size in patients who received rFVIIa but were unable to exhibit reduced mortality or improved functional outcomes after ICH (7).

There are limited data supporting the clinical role for rFVIIa in patients with DIC. There are reports of rFVIIa use in DIC patients with postpartum hemorrhage as well as those with trauma, sepsis, or cancer (8). One group has reported a case-series outlining the successful use of rFVIIa in patients with cancer and DIC-related bleeding complications (9). Also, Franchini and colleagues reported the successful use of rFVIIa in patients with critical hemato-oncologic bleeding associated with thrombocytopenia (10). Furthermore, Nosari and colleagues reported on the successful use of rFVIIa in an AML patient with ICH (11). Whether rFVIIa provides significant additional benefits to aggressive transfusion in patients with hematologic malignancies with ICH in the setting of severe thrombocytopenia and DIC remains unclear.

A recent study demonstrated that the use of rFVIIa for the prevention and treatment of bleeding in patients without hemophilia resulted in an increased incidence of arterial thromboembolic events (12). In a large and comprehensive cohort in placebo-controlled trials of rFVIIa, the risk of arterial thrombosis is associated with treatment with high doses of rFVIIa, especially among the elderly (13). Therefore, the routine use of high dose of rFVIIa in elderly patients without frequent evaluation of bleeding status is strongly discouraged.

Treatment of ICH with antifibrinolytic therapy including tranexamic acid, epsilon aminocaproic acid, or an equivalent resulted in significant reduction in the risk of re-bleeding in patients with aneurysmal subarachnoid hemorrhage (14).

It remains unclear whether the use of antifibrinolytic therapy resulted in shorter time to achieve hemostasis in patients with severe coagulopathy and thrombocytopenia.

Although current guidelines do not recommend the routine use of rFVIIa for the treatment of acute ICH (15), this case describes the safety and feasibility of a one-time administration of rFVIIa concurrent with continuous aminocaproic acid treatment in AML patients with life-threatening ICH. The optimal dose of and dosing schedule for rFVIIa are unclear, and further studies of this agent in patients with hematologic malignancies who experience life-threatening ICH refractory to aggressive transfusion support are warranted.

## Conflict of Interest Statement

The authors declare that the research was conducted in the absence of any commercial or financial relationships that could be construed as a potential conflict of interest.

## References

[1] Chen CY Tai CH Cheng A Wu HC Tsay W Liu JH Intracranial hemorrhage in adult patients with hematological malignancies. BMC Med (2012) 10:97. 10.1186/1741-7015-10-97 22931433 PMC3482556

[2] Uchiumi H Matsushima T Yamane A Doki N Irisawa H Saitoh T Prevalence and clinical characteristics of acute myeloid leukemia associated with disseminated intravascular coagulation. Int J Hematol (2007) 86:137–42 10.1532/IJH97.06173 17875527

[3] Brody DL Aiyagari V Shackleford AM Diringer MN. Use of recombinant factor viia in patients with warfarin-associated intracranial hemorrhage. Neurocrit Care (2005) 2:263–7. 10.1385/NCC:2:3:263 16159073 PMC2535929

[4] Rizoli SB Boffard KD Riou B Warren B Iau P Kluger Y Recombinant activated factor vii as an adjunctive therapy for bleeding control in severe trauma patients with coagulopathy: subgroup analysis from two randomized trials. Crit care (2006) 10:R178. 10.1186/cc4525 17184516 PMC1794494

[5] Creutzig U Ritter J Budde M Sutor A Schellong G. Early deaths due to hemorrhage and leukostasis in childhood acute myelogenous leukemia. Associations with hyperleukocytosis and acute monocytic leukemia. Cancer (1987) 60:3071–9. 10.1002/1097-0142(19871215)60:12<3071::AID-CNCR2820601235>3.0.CO;2-Y 3479232

[6] Mayer SA Brun NC Begtrup K Broderick J Davis S Diringer MN Recombinant activated factor vii for acute intracerebral hemorrhage. N Engl J Med (2005) 352:777–85 10.1056/NEJMoa042991 15728810

[7] Mayer SA Brun NC Begtrup K Broderick J Davis S Diringer MN Efficacy and safety of recombinant activated factor vii for acute intracerebral hemorrhage. N Engl J Med (2008) 358:2127–37. 10.1056/NEJMoa0707534 18480205

[8] Franchini M Manzato F Salvagno GL Lippi G. Potential role of recombinant activated factor VII for the treatment of severe bleeding associated with disseminated intravascular coagulation: a systematic review. Blood Coagul Fibrinolysis (2007) 18:589–93. 10.1097/MBC.0b013e32822d2a3c 17890943

[9] Sallah S Husain A Nguyen NP. Recombinant activated factor VII in patients with cancer and hemorrhagic disseminated intravascular coagulation. Blood Coagul Fibrinolysis (2004) 15:577–82. 10.1097/00001721-200410000-00008 15389125

[10] Franchini M Veneri D Lippi G. The potential role of recombinant activated fvii in the management of critical hemato-oncological bleeding: a systematic review. Bone Marrow Transplant (2007) 39:729–35. 10.1038/sj.bmt.1705670 17417659

[11] Nosari A Caimi TM Zilioli V Molteni A Mancini V Morra E Cerebral hemorrhage treated with novoseven in acute promyelocytic leukemia. Leuk Lymphoma (2012) 53:160–1 10.3109/10428194.2011.605189 21780994

[12] Simpson E Lin Y Stanworth S Birchall J Doree C Hyde C Recombinant factor VIIa for the prevention and treatment of bleeding in patients without haemophilia. Cochrane Database Syst Rev (2012) 3:CD005011 10.1002/14651858.CD005011.pub4 22419303 PMC11930396

[13] Yank V Stafford RS Safety of recombinant activated factor vii in randomized clinical trials. N Engl J Med (2011) 364(6):574–6 10.1056/NEJMc1013591#SA3 21306248

[14] Baharoglu MI Germans MR Rinkel GJ Algra A Vermeulen M van Gijn J Antifibrinolytic therapy for aneurysmal subarachnoid haemorrhage. Cochrane Database Syst Rev (2013) 8:CD001245 10.1002/14651858.CD001245.pub2 23990381 PMC8407182

[15] Morgenstern LB Hemphill JC III Anderson C Becker K Broderick JP Connolly ES Jr Guidelines for the management of spontaneous intracerebral hemorrhage: a guideline for healthcare professionals from the American heart association/American stroke association. Stroke (2010) 41:2108–29 10.1161/STR.0b013e3181ec611b 20651276 PMC4462131

